# Complete Genome Sequence of a Phenanthrene Degrader, *Mycobacterium* sp. Strain EPa45 (NBRC 110737), Isolated from a Phenanthrene-Degrading Consortium

**DOI:** 10.1128/genomeA.00782-15

**Published:** 2015-07-16

**Authors:** Hiromi Kato, Natsumi Ogawa, Yoshiyuki Ohtsubo, Kenshiro Oshima, Atsushi Toyoda, Hiroshi Mori, Yuji Nagata, Ken Kurokawa, Masahira Hattori, Asao Fujiyama, Masataka Tsuda

**Affiliations:** a Graduate School of Life Sciences, Tohoku University, Sendai, Japan; b Department of Computational Biology, Graduate School of Frontier Sciences, The University of Tokyo, Kashiwa, Japan; c Center for Information Biology, National Institute of Genetics, Mishima, Japan; d Department of Biological Information, Graduate School of Bioscience and Biotechnology, Tokyo Institute of Technology, Tokyo, Japan; e Earth-Life Science Institute, Tokyo Institute of Technology, Tokyo, Japan; f Principles of Informatics Research Division, National Institute of Informatics, Tokyo, Japan

## Abstract

A phenanthrene degrader, *Mycobacterium* sp. EPa45, was isolated from a phenanthrene-degrading consortium. Here, we report the complete genome sequence of EPa45, which has a 6.2-Mb single circular chromosome. We propose a phenanthrene degradation pathway in EPa45 based on the complete genome sequence.

## GENOME ANNOUNCEMENT

Many polycyclic aromatic hydrocarbons (PAHs) are serious environmental pollutants, and their toxicity threatens human and wildlife health (1, 2). Microbial biodegradation of PAHs has thus drawn considerable attention, and various PAH-degrading bacterial strains have been isolated (3).

In our previous laboratory study, a soil sample was artificially polluted with four aromatic hydrocarbons—phenanthrene, biphenyl, carbazole, and 3-chlorobenzoate (4). Fifteen weeks after the pollution, a portion of the soil sample was inoculated into 1/10-strength W minimal liquid medium (5) supplemented with 0.5 mM phenanthrene as a carbon source. After cultivation with shaking at 30°C for 2 weeks, a portion of the culture was diluted and spread on the same medium supplemented with 1.5% agar. Incubation for 10 days led to the formation of a colony of a phenanthrene-degrading microbial consortium, designated MixEPa4. Our repeated single-colony isolation of MixEPa4 resulted in the isolation of a pure phenanthrene-degrading strain, *Mycobacterium* sp. EPa45. (This strain has been deposited in the NITE Biological Resource Center [NBRC, Japan] under code NBRC 110737.) The relative compositions of *Mycobacterium* (5%) and the other major proteobacterial genera in MixEPa4 were stably maintained in the minimal liquid culture at least during the period of phenanthrene degradation. To investigate the mechanism(s) underlying the stable maintenance of the community members in the future, we determined in this study the complete genome sequence of *Mycobacterium* sp. EPa45.

The 454 GS FLX Titanium (Roche) and MiSeq platforms (Illumina) were used for sequencing of the EPa45 genome. The 454 shotgun and paired-end sequencing generated 180,512 reads (71 Mb) and 85,515 reads (29 Mb), respectively. The reads obtained by MiSeq paired-end sequencing of a Nextera mate pair library were trimmed based on underrepresented 21-mers by our ShortReadManager program (6). The trimming produced 3,515,328 reads (447 Mb). The resulting reads were assembled using Newbler software version 3.0 (Roche), producing 282 contigs and a single scaffold with 54 gaps. The finishing was facilitated by our GenoFinisher and AceFileViewer programs (6) (http://www.ige.tohoku.ac.jp/joho/gf_e); our *in silico* analysis closed 31 gaps, and PCR amplification and Sanger sequencing were conducted to close 23 gaps. The finished sequence was confirmed by FinishChecker, a tool of GenoFinisher (6). The NCBI Prokaryotic Genomes Annotation Pipeline (http://www.ncbi.nlm.nih.gov/genome/annotation_prok) was used to identify and functionally annotate the rRNA, tRNA, and protein-coding genes.

The complete genome of EPa45 consists of a 6,177,406-bp single circular chromosome with 66% G+C content and carries two copies of rRNA operons, 47 tRNA genes, and 5,599 protein-coding sequences. Functional annotations suggested that phenanthrene is degraded to the TCA cycle compounds by the pathway via the key intermediates, *o*-phthalate, protocatechuate, and β-ketoadipate (7, 8). Information on the EPa45 genome sequence will greatly contribute to our understanding the mechanism(s) for the stable maintenance of microbial community members in MixEPa4 during the phenanthrene degradation.

### Nucleotide sequence accession number.

The complete genome sequence of *Mycobacterium* sp. EPa45 has been deposited at GenBank under the accession number CP011773.
